# The Influence of Inset and Shaping of Abdominal-Based Free Flap Breast Reconstruction on Patient-Reported Aesthetic Outcome Scores—A Systematic Review

**DOI:** 10.3390/jcm13082395

**Published:** 2024-04-19

**Authors:** Isabel Zucal, Laura De Pellegrin, Corrado Parodi, Yves Harder, Riccardo Schweizer

**Affiliations:** 1 Department of Plastic, Reconstructive and Aesthetic Surgery, Ospedale Regionale di Lugano, Ente Ospedaliero Cantonale (EOC), Via Capelli 1, 6900 Lugano, Switzerland; isabel.zucal@eoc.ch (I.Z.); laura.depellegrin@eoc.ch (L.D.P.); corrado.parodi@eoc.ch (C.P.); yves.harder@eoc.ch (Y.H.); 2 Faculty of Biomedical Sciences, Università della Svizzera Italiana (USI), Via Buffi 13, 6900 Lugano, Switzerland; 3 Department of Hand Surgery and Plastic Surgery, Luzerner Kantonsspital, Spitalstrasse, 6000 Lucerne 16, Switzerland

**Keywords:** aesthetics, breast, breast reconstruction, free flap, patient satisfaction

## Abstract

**Background:** Nowadays, multimodal cancer therapy results in very high survival rates of early-stage breast cancer and microsurgical flap-based breast reconstruction has become safe and reliable, with gradually increasing demand because of its durable and aesthetically pleasing results. This study aimed to explore the impact of different flap shaping and inset techniques on patient-reported outcome measures (PROMs) with regard to the aesthetic result in abdominal flap-based breast reconstruction. **Methods:** A systematic review was performed screening Pubmed, Cochrane Library and Web of Science for original articles reporting flap inset strategies, concomitantly providing PROMs on the aesthetic result. **Results:** Of 319 studies identified, six met the inclusion criteria. The studies described different flap rotation options according to the patient’s morphology, different inset planes, and avoidance of the monitoring skin paddle, and suggested that a higher flap-to-mastectomy mass ratio was associated with better aesthetic results. In two comparative studies, according to the PROMs (BREAST-Q, Likert scale) and independent observer judgement, both higher patient satisfaction and superior aesthetic results were observed with the newly described techniques. **Conclusions:** Emphasis on the aesthetic outcome in terms of breast shape and symmetry, providing an individualized approach of flap inset, considering the contralateral breast’s shape and volume, results in higher satisfaction scores.

## 1. Introduction

Increased awareness for preventive breast check-ups [1,2], advancements in screening techniques for breast cancer [1,2,3] and effective multimodal treatment—both in a neoadjuvant and adjuvant setting—[1,4,5,6] have ensured that early-stage breast cancer has nowadays an excellent survival rate. Accordingly, aesthetic aspects of the reconstructed breast have significantly gained importance, particularly in the light of increasing rates of mastectomies performed in rather young and healthy gene carriers and high-risk patients [7,8,9].

Reconstructions need not only to be definitive and durable, but also natural in shape and haptics [9]. Furthermore, the lowest possible morbidity has become another issue in breast reconstruction requiring autologous tissue, such as flap-based or hybrid breast reconstruction [10]. Following mastectomy—therapeutic or prophylactic—there are different reconstructive options, including implants, non-vascularized autologous fat injected as a graft as well as vascularized autologous tissue used as a flap. These techniques allow individualized and tailored breast reconstruction depending on the patient’s anatomical characteristics, needs and expectations [9,11].

Although more invasive and associated with “collateral” damage at the donor site due to flap harvesting [10], autologous flap-based breast reconstruction can provide very good aesthetic results, avoiding potential long-term breast implant-related complications, such as capsular contracture, implant migration, implant rupture, breast implant-associated anaplastic large cell lymphoma or breast implant illness [12,13]. Flaps can be harvested from different donor sites of the body, though the abdominal adipo-cutaneous tissue excess based on the deep inferior epigastric vessels is most commonly used due to its rather frequent presence. 

To fulfill these requirements, proper shaping and inset of the flap—most often in a pre-defined mastectomy pocket following skin-sparing mastectomy or skin expansion—is gaining importance. Several techniques have been described, considering the type of donor site, volume and skin to be replaced, laterality and anatomical characteristics of the source vessels and their perforators, as well as the recipient vessels. However, there is only a small body of scientific evidence regarding the aesthetic outcome of flap-based breast reconstruction that emphasizes flap inset and shaping techniques [14]. Even less represented are studies evaluating patient-reported outcome measures (PROMs) with regard to the aesthetic satisfaction in abdominal flap-based breast reconstruction depending on the shaping and inset techniques. In fact, in order to objectively compare surgical techniques and record the patient’s subjective perceptions, PROMs are paramount [15,16].

Herein, the study’s aim was to review the current literature regarding the impact of flap shaping and inset on PROMs including aesthetic outcome scores in abdominal flap-based breast reconstruction.

## 2. Materials and Methods

### 2.1. Literature Search Strategy

Pubmed, Cochrane Library and Web of Science databases were screened. The search terms were: “diep (deep inferior epigastric perforator) flap AND insetting”, OR “ms tram (transverse rectus abdominis muscle) AND insetting”, or “diep flap AND orientation”, OR “ms tram AND orientation”, OR “diep flap AND shaping”, OR “ms tram AND shaping”. Screening with these terms was performed on 23 August 2023. Studies analyzing aesthetic outcomes and containing a personalized questionnaire such as the BREAST-Q or a comparable questionnaire were included. Case reports and articles not written in English were excluded. A review protocol was developed based on the Preferred Reporting Items for Systematic Reviews and Meta-Analyses (PRISMA) statement (www.prisma-statement.org (accessed on 23 August 2023)). This review has not been registered.

### 2.2. Data Extraction

Two independent reviewers (I.Z. and R.S.) screened all titles and abstracts. After excluding all duplicates, the full texts were screened for eligibility according to the inclusion and exclusion criteria. In case of disagreement between the two reviewers, a third reviewer (Y.H.) was consulted in order to reach a consensus. The following data were registered on an electronic table in Excel (Microsoft, version 16.83, (24031120)): year and author of the article, study design, number of patients included, inset/shaping method of the abdominal flap, type of questionnaire (PROM type), time point of PROM evaluation, presence of control group, outcome, mastectomy laterality and type, radiation therapy, contralateral mastopexy/reduction mammoplasty, immediate or delayed reconstruction, and aesthetic outcome score.

### 2.3. Assessment of Risk of Bias and Quality of Evidence of the Included Studies

To assess the quality of the comparative studies included, the Downs and Black checklist was used [17]. The checklist contains 27 questions on the domains: reporting, external validity, internal validity, and power of the study. The checklist was completed independently by two authors (I.Z. and R.S.) and a third author (Y.H.) was consulted to reach a consensus in case of discrepancies.

### 2.4. Statistical Analysis

Results were reported descriptively. 

### 2.5. Graphical Illustration

In our graphical illustrations, Holm zones [18] were used to provide consistent and easily understandable figures, even if there was no specific mention of the perfusion zones or mention of the Hartrampf zones in the cited papers.

## 3. Results

### 3.1. Literature Search Results

**Figure 1:**
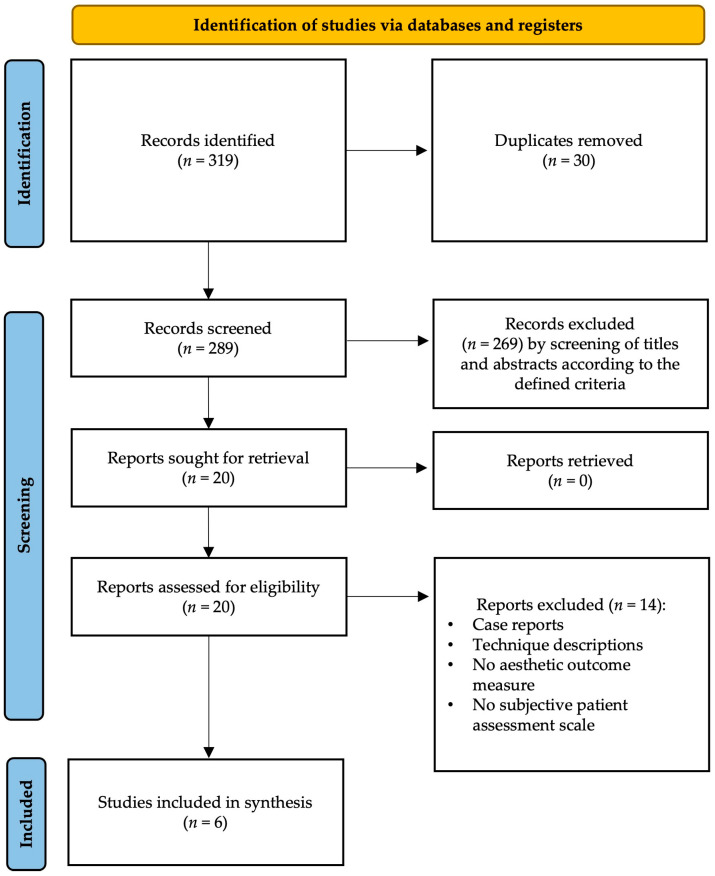
PRISMA diagram of the screening process and included papers.

A total of 319 articles were identified using the defined terms. After screening all titles and abstracts, 30 duplicates were removed, resulting in 289 records. Further evaluation (screening of titles and abstracts) led to an exclusion of 269 records: studies not pertinent to the topic and not corresponding to the determined inclusion/exclusion criteria were excluded (inclusion of studies reporting aesthetic outcomes and patient-reported outcome measures such as the BREAST-Q; exclusion of case reports and articles not written in English). Twenty studies were defined as eligible. Analysis of these full-text articles resulted in the inclusion of six studies according to the defined inclusion and exclusion criteria. An overview of the screening process is provided in Figure 1. An overview of the included studies is summarized in Table 1.

Due to the lack of comparative studies (*n* = 2), matchable follow-up periods, differences in the outcome scales, and quality of the studies, it was not possible to perform any metanalysis. Therefore, data were presented descriptively.

### 3.2. Study Details

A total of 271 female patients were included in this review. In the first study of Gravvanis et al., a prospective comparative study was carried out with 25 patients undergoing flap insertion in a subpectoral, dual plane compared to 25 patients undergoing flap insertion in a prepectoral, single plane [19]. In their second study, Gravvanis et al. prospectively analyzed 42 patients with a dual plane inset of the flap [20]. The PROMs were recorded via the Likert scale in both studies: a questionnaire with six questions that can be scored with 0–4 points. Moreover, in the first study, 30 independent evaluators scored the aesthetic outcome on a visual analogue scale from 0–100 mm.

Razzano et al. described the prospective evaluation of 70 consecutive immediate unilateral DIEP flaps with an inset based on the author’s algorithm [21]. This algorithm considered donor site properties (i.e., adipose tissue proportion of the donor site), as well as the contralateral breast’s characteristics (including the breast’s footprint, volume and shape) for decision-making regarding flap-inset. The PROMs were represented by means of a BREAST-Q (scale from 0–100). Moreover, the aesthetic outcome (breast projection, shape, volume, symmetry and ptosis) was evaluated by three independent evaluators (surgeon, nurse and secretary).

Francis et al. performed a prospective comparative study, including 12 patients who underwent breast reconstruction with a DIEP flap, which was completely de-epithelialized, buried under the mastectomy flap, avoiding the monitoring skin paddle leaving “open” a small “observation window” at the incompletely closed skin incision at the inframammary fold for flap monitoring after positioning of retention sutures [22]. This “window” was then closed post-primarily tightening the retention sutures after the monitoring period of the flap was over [22]. The aesthetic outcome of these patients, evaluated by a BREAST-Q, was compared to 12 patients undergoing breast reconstruction with a DIEP flap with the monitoring skin paddle [22]. Moreover, a Manchester scar scale was completed by seven plastic surgeons/fellows (the lower the score, the better the aesthetic outcome).

Long et al. retrospectively reviewed the BREAST-Qs of a series of 45 cases who underwent abdominal flap-based breast reconstruction and analyzed whether a higher flap-mass-to-mastectomy-mass ratio was associated with higher patient satisfaction [23]. 

Finally, Dung et al. just recently reviewed the BREAST-Qs of 40 cases with an oblique inset of the DIEP flap retrospectively without any control group [24]. 

### 3.3. Inset and Shaping Techniques

**Figure 2:**
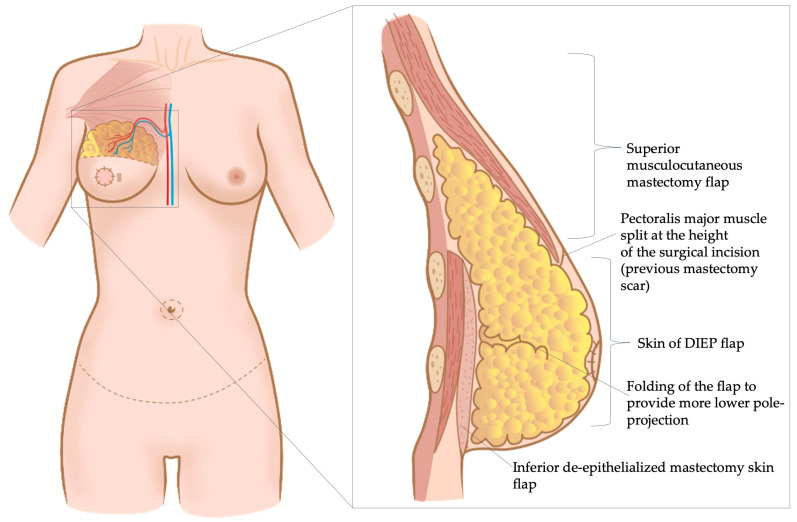
Dual plane flap inset technique: Incision of skin and muscle is performed at the level of the mastectomy scar and the flap is buried underneath the cranial part of the pectoralis major muscle. The skin inferior to the mastectomy scar is de-epithelialized and the caudal part of the abdominal flap is placed above the de-epithelialized skin to reconstruct the lower pole of the breast [19,20].

In the study of Gravvanis et al., all the included patients underwent unilateral, radical-modified mastectomy [19,20]. For secondary breast reconstruction, an incision was made at the level of the mastectomy scar and the skin between the incision and the inframammary fold, i.e., the distal mastectomy flap was de-epithelialized [19,20]. Thereafter, the pectoralis major muscle was split at the level of the incision (i.e., previous mastectomy scar), creating a submuscular pocket cranially to this musculocutaneous flap [19,20]. The DIEP-flap was then inserted behind the pectoralis major muscle in the cranial part and above the muscle in the caudal part, onto the de-epithelialized mastectomy skin [19,20] (Figure 2).

**Figure 3:**
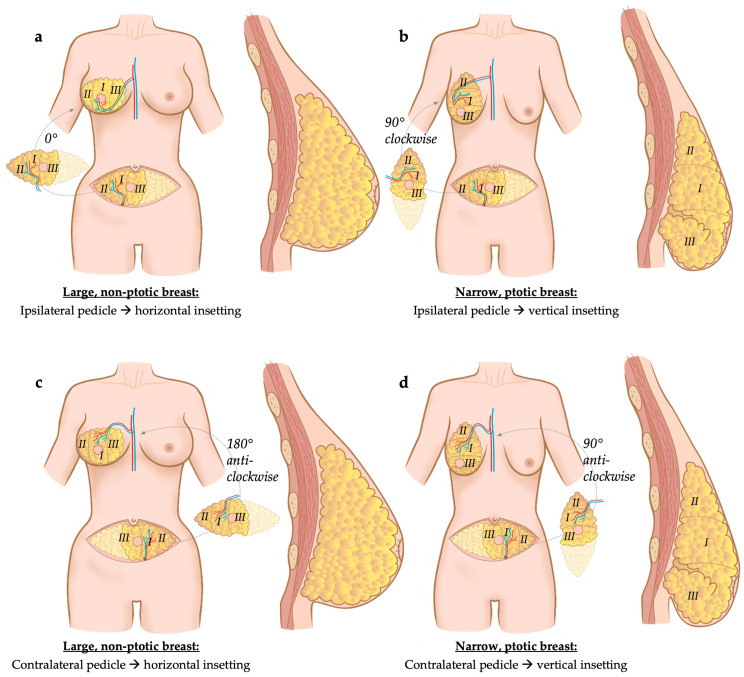
Flap inset according to breast shape and volume: (**a**) If flap elevation including pedicle preparation is performed on the ipsilateral side (i.e., the side of the breast to be reconstructed) in patients with a large, non-ptotic breast, flap inset is performed in a horizontal way without rotation. (**b**) If flap elevation is performed on the ipsilateral side in patients with a narrow and/or ptotic breast, breast reconstruction requires flap rotation by 90° clockwise to allow vertical inset. (**c**) If flap elevation is performed on the contralateral side in patients with a large, non-ptotic breast, the inset requires flap rotation by 180° anti-clockwise to be placed horizontally. (**d**) Finally, if flap elevation is performed on the contralateral side in patients with a narrow and/or ptotic breast, reconstruction requires flap rotation by 90° anti-clockwise to allow a vertical inset. The vessels of the flap’s pedicle are anastomosed to the internal mammary artery and vein(s) [21]. Holm zones (I–III) are shown in the figure.

In Razzano et al., donor site characteristics such as thick versus thin abdominal wall thickness were considered, as well as the shape of contralateral breast (ptotic/narrow footprint versus projected/large footprint), number and position of vascular perforators originating from the source vessel (deep inferior epigastric artery and vein, uni- versus bipedicled flap, ipsi- versus contralateral flap with regards to breast to be reconstructed) and of the caliber of the superficial inferior epigastric vessels [21]. Based on these factors, the flap was either oriented vertically or horizontally during its inset [21]. For instance, in slim patients with a ptotic contralateral breast (≥2nd degree according to Regnault’s classification [25]), a vertical inset of the flap was preferred, rotating it by 90° clock- or anti-clockwise, depending on the laterality of the pedicle [21]. In patients with thicker abdominal soft tissues (subcutaneous fat thickness exceeding 2.5 cm) and in patients with a projected breast, flap inset was rather performed in a horizontal way, without rotation or by rotating the flap by 180°, depending on the laterality of the pedicle [21] (Figure 3). Bi-pedicled flaps were used if more than 70% of the lower abdominal tissue was required to match the contralateral breast’s volume, i.e., perfusion of all four Holm zones was needed or if cross-midline perfusion was impaired in a flap requiring more than two zones. When bi-pedicled flaps were used, both pedicles of the flap were anastomosed to the internal mammary vessels, one proximally in an antegrade way, and the second one distally in a retrograde way. If needed, contralateral symmetrization of breast volume and shape was performed simultaneously [21]. 

**Figure 4:**
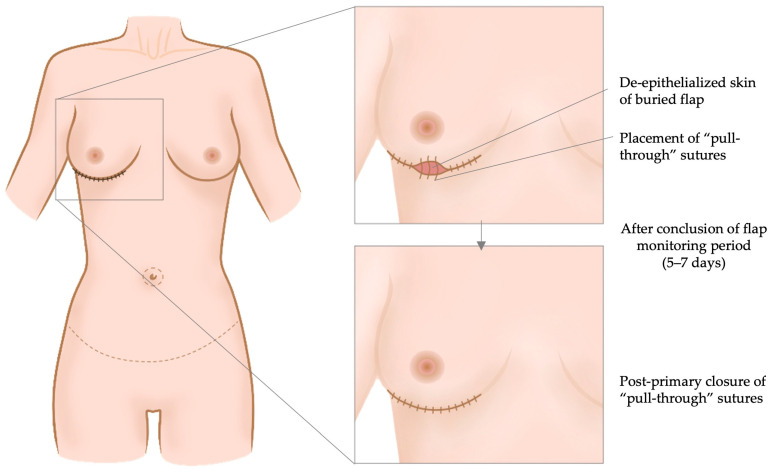
The abdominal flap is completely de-epithelialized and buried underneath the mastectomy skin. The mastectomy skin is then closed above the flap except for a small “window”, allowing for direct flap monitoring. Transcutaneous sutures are already placed in order to re-approximate the skin margins by tightening the sutures (post-primary retention sutures), once the observation period is over (after 5–7 days). This allows the avoidance of post-primary excision of a monitoring skin paddle [22].

Francis et al. aimed to prevent any use of skin paddles for monitoring in order to avoid subsequent surgery for its removal [22]. The DIEP flap’s pedicle was always anastomosed laterally, to the thoracodorsal vessels [22]. Thereafter, the flap was completely de-epithelialized and a flap area of approximately 3 × 1 cm^2^ was anchored using “pull through” retention sutures to the skin margins of the mastectomy incision [22]. This allowed visualization of a small de-epithelialized flap area for its monitoring (i.e., color, capillary refill, pinch bleeding). This monitoring window was medicated at needs with wet gauzes [22]. Following the monitoring period of 5–7 days, the positioned sutures could be tightened to achieve post-primary wound closure (Figure 4).

Long et al. evaluated the BREAST-Q scores with regard to the flap-to-mastectomy mass ratio, hypothesizing that a ratio > 1:1 was associated with better scores in patient-reported outcomes [23].

**Figure 5:**
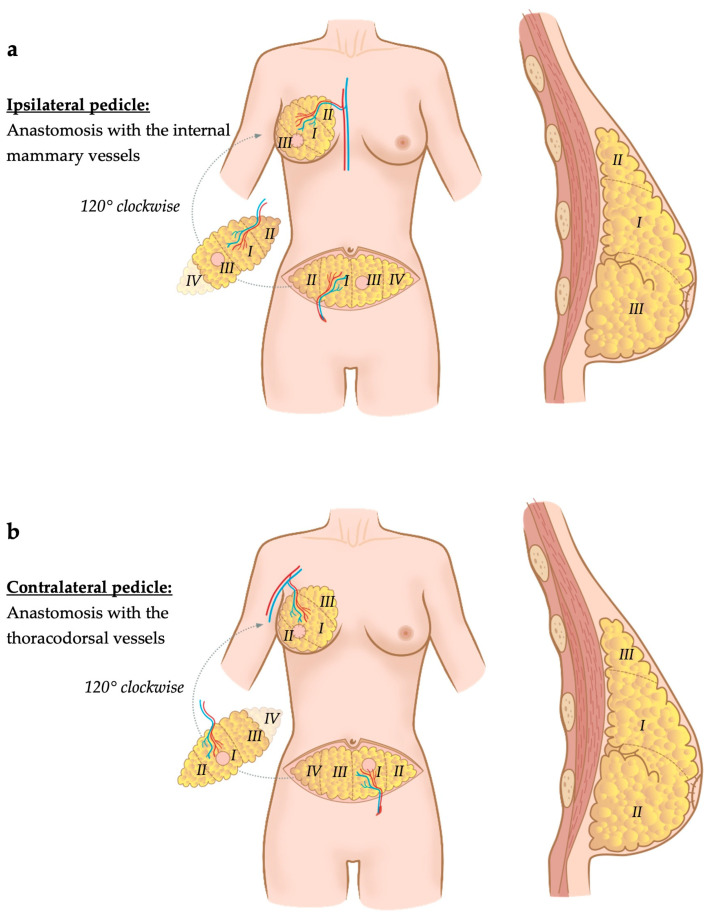
Oblique flap inset: Regardless of the shape of the contralateral breast, the flap inset is oblique. (**a**) If the flap’s pedicle is elevated on an ipsilaterally located perforator regarding the breast to be reconstructed, the flap is rotated by 120° clockwise and the pedicle is anastomosed medially to the internal mammary vessels. (**b**) If the perforator originates from the flap’s pedicle on the contralateral side, the flap is rotated 120° clockwise and the pedicle is anastomosed laterally to the thoracodorsal vessels [24]. Holm zones (I–IV) are visualized in the figures.

Finally, Dung et al. described an oblique inset technique of the DIEP flap [24] in immediate breast reconstruction after mastectomy. Water displacement was used to measure the volume of flap tissue needed for reconstruction. The authors decided to use an approximately 10% higher flap volume than the mastectomy volume, though the reason for this is not explained in the paper. After flap tailoring to reach adequate volume, the flap was rotated by 120° clockwise and the thicker peri-umbilical margin of the flap was placed infero-medially to provide more volume and eventually more projection, whereas the pubic margin of the flap was placed supero-laterally [24]. If the pedicle that was used to perfuse the flap was harvested contralateral to the breast to be reconstructed, the flap’s pedicle was anastomosed to the thoracodorsal vessels, whereas if it originated from the ipsilateral side, the internal mammary vessels served as recipient vessels [24]. After performing the anastomosis, a fixation suture was put cranially at the intercostal space of the 2nd and 3rd rib. Eventually, the skin was closed intercalating a monitoring skin paddle (Figure 5). 

### 3.4. Outcome

Gravvanis et al. reported as follows in their comparative study including 50 patients with a mean follow-up time of 29 months in both groups [19]: 26 of 30 independent evaluators favored the dual plane group [19]. Significantly higher scores were given in five of seven aesthetic outcome questions by the evaluators [19]. In the PROMs (Likert scale), a significantly higher score was achieved in the dual plane group in the questions about “satisfaction without brasserie”, “fullness of the upper pole” and “ptosis of breast with time” [19]. In fact, in the single-plane prepectoral group, five patients requested flap revision to correct ptosis over time, whereas no patient in the subpectoral dual plane group underwent corrective surgery [19]. No significant difference was observed in the questions about “effects on social life” and “effects on sexual life” between the groups [19]. These aesthetic outcome measures in favor of the dual plane technique were confirmed in the second study of Gravvanis et al. with a long-term follow-up of 2 years [20].

Razzano et al.’s individualized inset algorithm was evaluated by BREAST-Q at 12 months after reconstruction [21]. Patients reported a mean overall score of 82/100 [21]. The highest scores were observed for the questions about “satisfaction with the surgeon, medical staff and office staff”, whereas the lowest scores were seen in “sexual well-being” [21]. Interestingly, complications requiring surgical revision did not affect the overall BREAST-Q score. However, ischemia-related fat necrosis within the flap reduced the reported satisfaction with the breasts and psychological well-being [21]. In addition, 51 of the 70 patients completed a pre-and postoperative photo session and three independent evaluators assessed the pictures [21]. The scores revealed mostly “good” or “very good” results [21]. Disagreement was found among the evaluators with regard to breast symmetry and ptosis, whereas agreement was observed for the questions about projection, shape and volume [21].

In the comparative study of Francis et al., the BREAST-Q was completed at 9 months after surgery, showing that the post-primary closure yielded more satisfaction with the reconstructed breast compared to the patients with the monitoring skin paddle (68 vs. 62/100 points) [22]. At 12 months follow-up, the Manchester scar scale displayed a significant difference in the questions about “scar distortion”, “scar visual analogue scale” and “overall scale” favoring the post-primary retention suture group [22].

In the retrospective analysis of Long et al., 45 patients evaluating a total of 70 reconstructed breasts were included with a 64.2% BREAST-Q survey response rate [23]. The mean time to survey completion was 21 months after breast reconstruction [23]. A total of 40% of the included patients underwent radiation therapy [23]. BREAST-Q evaluation revealed that a higher flap-to-mastectomy mass ratio (flap mass 26% higher than mastectomy mass) was associated with better scores in the question about “satisfaction with the breasts” [23]. An overall BREAST-Q score of 65/100 was reached [23]. However, no significant differences were found in the questions about “physical well-being of the chest” or “abdominal well-being”, “sexual well-being”, and “psychosocial well-being” [23]. Radiation therapy correlated negatively with reported satisfaction with the breasts [23].

Finally, Dung et al. evaluated the BREAST-Qs at 6 months and found an average overall satisfaction score of 62/100 [24]. It has to be mentioned that this score was markedly lower compared to the score reported by Razzano et al. (62/100 vs. 82/100) [21]. Patient characteristics, surgery-specific data and aesthetic outcome scores are presented in Table 2.

## 4. Discussion

A high satisfaction concerning aesthetic outcome scores was demonstrated using different flap-inset techniques. In fact, due to continuous refinements in equipment and microsurgical techniques, free flap surgery for autologous breast reconstruction has reached a very high flap survival rate [26,27]. Consequently, much more effort is now put into durability and aesthetic outcome of breast reconstruction, focusing not only on shape, symmetry and volume of the breast if needed, but also on donor site morbidity and appearance, including positioning of the scars [28].

All studies included in this review describe rather inset than shaping techniques and flap dimensions, but no studies focusing on the shaping of the flap only were found. However, inset and shaping are linked to each other, and the inset of the flap somehow determines its shape. 

Among the herein-presented studies, Razzano et al. reached the highest patient satisfaction scores using a personalized approach with individualized flap orientation and inset [21]. Flap orientation according to the contralateral breast’s shape has been supported in literature with regard to aesthetic outcomes [29]. For an optimal inset of the rotated flap, pedicle length and orientation need to be considered [30]. On the other hand, Dung et al. described an oblique flap inset regardless of the factors considered by Razzano et al. However, this inset technique proposed by Dung et al. was associated with lower aesthetic satisfaction scores of approximately 20% compared to Razzano et al. Furthermore, the flap failure rate of 7% was rather high compared to the current literature [21,24]. In the case of flap preparation using a contralateral pedicle, Dung et al. propose a clockwise rotation of 120° and use the thoracodorsal vessels in the axilla as the recipient vessels [24]. This results in the positioning of the potentially less well-perfused Holm zone III of the flap in the cleavage area. According to our personal experience, an approximately 45° anti-clockwise rotation of the flap allows to inset the flap obliquely, with the less thick ipsilateral zone II in the upper outer quadrant of the reconstructed breast and the cleavage area and the thicker periumbilical zone I in the central area. In order to further increase the lower pole volume and eventual projection of the breast, Holm zone III can be folded, i.e., the zone beyond the flap’s midline (more at risk for fat necrosis, particularly if the flap is based on one pedicle). Accordingly, fat necrosis would not occur in the cleavage area, but rather in the lower and outer region of the reconstructed breast. Since zone IV is usually discarded when using only a single-pedicled DIEP flap, we do not discuss in detail the possibilities of how safely all four zones of the flap can be preserved and insetted.

Nowadays, a pre-pectoral plane is chosen for flap insertion in almost all autologous breast reconstructions. Gravvanis et al., however, challenged this approach and described a dual plane technique with the cranial part of the flap inserted under the pectoral muscle with a high patient satisfaction regarding the aesthetic appearance of the breast when compared to a matched cohort with a pre-pectoral flap inset [19,20]. Francis et al. provided an innovative technique for immediate scar reduction by performing post-primary wound closure using retention sutures [22], contributing to an aesthetically pleasing result which reflected in high patient satisfaction scores and avoiding secondary surgery for excision of the monitoring skin-island. 

In literature, many authors describe their personal techniques on flap shaping, inset and orientation, but larger case series, as well as prospective, or even randomized comparative studies between different techniques reporting PROMs on the aesthetic outcomes, are strongly lacking.

For instance, a “Calzone” flap technique to reconstruct medium to large-sized breasts has been described by raising a DIEP flap using both DIE-artery pedicles [31]. This allows to use of all four Holm zones of the flap to be insetted in a rather vertical way and folded at its midline [31]. Then, the pedicles are anastomosed to the internal mammary vessels, both proximally in an anterograde manner and distally in a retrograde manner [31]. This technique aims at guaranteeing the best perfusion of a flap that becomes two times a “half” flap including Holm zones I and II each instead of one entire flap including Holm zones I–IV. Accordingly, the risk of fat necrosis is significantly reduced. Further, by placing the fold of the flap infero-laterally, inferior bulk and eventually breast projection and a natural shape are provided [31]. In a case series of Odobescu et al., a similar flap shaping technique has been described: two sutures were positioned at 10 and 2 o’clock and ran at the level of the 6 o’clock-position folding the flap and providing projection [32]. Accordingly, bi-pedicled DIEP flaps were suggested to guarantee better perfusion in more voluminous flaps [33,34]. 

In contrast, to harvest less tissue at the donor site and allow for better donor site closure with less wound healing problems, Patel et al. described an inverted, deepithelialized V-shaped flap originating from the distal adipocutaneous mastectomy flap in order to gain volume in the inferior quadrants of the reconstructed breast [35]. Accordingly, Hamdi et al. described the “hug flap” technique for patients with insufficient thickness of the abdominal wall tissue or when high breast projection is desired: the caudal mastectomy skin is de-epithelialized and the lateral and medial skin flaps are undermined and folded over the central part [36]. This way, the infero-central volume increases, providing more projection, somehow similar to a central mound or “auto-augmentation” used when reshaping a sagged breast in order to restore infero-central volume [36].

Jeong et al. reviewed 274 patients undergoing either vertical or horizontal inset of TRAM or DIEP flaps [37]. A total symmetry score was calculated and they found that vertical inset was associated with higher total symmetry scores, projection and ptotic naturalness scores [37]. Moreover, with a vertical inset of the flap, more symmetrical volume distribution in the upper, medial and lateral poles were achieved [37].

Similar to Dung et al., many authors tried to avoid multiple steps in breast reconstruction to provide an immediate and aesthetically pleasing breast reconstruction [24]. In this regard, Laporta et al. performed immediate surgery of the contralateral breast in 31 patients undergoing DIEP flap-based breast reconstruction in order to achieve symmetry and compared it to 17 cases with a two-stage procedure [38]. Operative time was 37 min longer in the one-stage procedure group. The aesthetic results were rated by the patients and two blinded plastic surgeons based on pre- and postoperative photographs, showing no differences in volume, shape, projection, and general satisfaction scores compared to the two-stage approach [38]. 

In a further study by El Khatib et al., a wise pattern mastectomy was suggested before immediate DIEP flap breast reconstruction, allowing for immediate skin reduction and shaping of the breast [39]. However, in 5 out of 53 cases (9.4%), a significant mastectomy flap necrosis requiring surgical revision was described [39].

To achieve satisfying aesthetic results, intermammary symmetry is very important. In this regard, different modalities that determine the required flap volume have been described, including pre- and intraoperative 3D scanning, water displacement of the mastectomy specimen or 3D-printed scaffolds of the breast to be operated [40,41]. Tomita et al. suggested the use of an inverted 3D-printed mold of the contralateral breast and satisfactory aesthetic results were achieved postoperatively based on 3D measurements of both breasts [41,42]. Tregaskiss et al. also described a preoperative template and molding technique in order to preoperatively plan the amount of DIEP flap tissue to be harvested and to plan the skin paddle, increasing the efficacy of the surgical procedure and the aesthetic results [43].

One limitation allowing an objective comparison of the aesthetic result of different surgical techniques is that the judgement of beauty “is in the eye of the beholder” and therefore is influenced by subjective experiences and perceptions. Moreover, the aesthetic result is significantly affected by culture, ethnicity and sociodemographic factors. In this regard, Raposio et al. demonstrated that the consideration of the body’s shape determines the perception of beauty of the size of the breast. Furthermore, the judgement of the attractiveness of a breast is influenced by factors such as age, the role of sex and marital status [44]. Accordingly, Razzano et al. could well demonstrate that the consideration of the body’s shape results in high aesthetic satisfaction scores [21].

In summary, different techniques are described focusing on shaping, orientation, inset and fixation of the flap by sutures, mainly depending on the experience of the senior surgeon who performs most cases. This leads to the high heterogeneity of all described techniques reported in the case series, but studies reporting PROMs and comparative studies including the above-mentioned variables are lacking.

## 5. Conclusions

Individualized inset and shaping techniques that consider donor and recipient site characteristics, including volume and shape of the contralateral breast provide higher patient satisfaction and superior aesthetic outcomes according to the reported PROMs. The choice of the surgical technique in general and the way the flap is inset and shaped in particular is also very often surgeon-dependent and determined by the familiarity of the surgeon with surgical technique, as well as her/his personal perception of breast beauty.

Large, comparative studies with standardized outcome scales on this specific topic are lacking, providing insufficient evidence on the aesthetic superiority of one of the described techniques over the others. 

## Figures and Tables

**Table 1:** Included studies and study characteristics.

Article (Year, Author)	Study Design	Patients (*n*)	Inset/Shaping	Questionnaire (PROM)	Time-Point of Evaluation	Presence of Control Group	Outcome
2015, Gravvanis et al. [19]	prospective, comparative	50	group A (*n* = 25): flap inset using prepectoral single plane; group B (*n* = 25): flap inset using dual plane (pre- and subpectoral)	Likert scale	not specified	Yes (prepectoral, single plane)	patient self-evaluation, aesthetic outcome evaluated by 30 evaluators (7 questions based on postoperative photographs and VAS (0–100 mm))
2016, Gravvanis et al. [20]	prospective	42	dual plane inset	Likert scale	2 and 24 months	none	patient self-evaluation
2019, Razzano et al. [21]	prospective	70	inset upon the author’s algorithm depending on patients’ morphology, contra- or ipsilateral breast, uni- or bipedicled flap, shape and volume of the contralateral breast	BREAST-Q	12 months	None	patient self-evaluation), aesthetic outcome evaluated by three independent assessors
2022, Francis et al. [22]	prospective, comparative	24	post-primary retention suture after burying the DIEP flap instead of using a skin paddle	BREAST-Q	9 months	yes (monitoring skin paddle)	patient self-evaluation, Manchester scar scale (the lower the score, the better the cosmetic outcome) completed by seven plastic surgeons/fellows
2023, Long et al. [23]	retrospective	45	higher flap-to-mastectomy mass ratio	BREAST-Q	21 months	none	patient self-evaluation
2023, Dung et al. [24]	retrospective	40	oblique inset, flap volume 10% > mastectomy volume (water displacement)	BREAST-Q	6 months	none	patient self-evaluation

PROM = patient-reported outcome measure; VAS = visual analogue scale.

**Table 2:** Patients’ characteristics and aesthetic outcome scores of the included studies.

Article (Year, Author)	Mastectomy laterality and Type (*n*)	Radiation Therapy	Contralateral Mastopexy/Reduction Mammoplasty (*n*)	Immediate vs. Delayed Reconstruction	Aesthetic Outcome Score
2015, Gravvanis et al. [19]	Unilateral, radical modified (50/50)	50/50, adjuvant	Intervention group (dual plane, 17/25) Control group (pre-pectoral, 18/25)	Delayed	Likert scale completed by 17/25 patients in the prepectoral plane group and 16/25 patients in the dual plane group: significant difference in favor of the dual plane group in the questions about “satisfaction without brasserie”, “ptosis of breast with time” and “fullness of the upper pole” VAS evaluated by 30 external observers: significant difference in favor of dual plane in the questions about “overall breast appearance”, “superior scar”, “superior mastectomy skin”, “natural transition”, “outline of the breast”; 26/30 evaluators preferred dual plane group
2016, Gravvanis et al. [20]	Unilateral, radical modified (42/42)	42/42, adjuvant	26/42	Delayed	Likert scale completed by 42/42 patients: no significant difference between 2-month and 2-year follow-up, especially high scores in the questions about “satisfaction with brasserie”, “satisfaction without brasserie”, and “fullness of the upper pole” at the 2-year follow-up
2019, Razzano et al. [21]	Unilateral, both skin-sparing (64/70) and non-skin-sparing (6/70)	Not reported	29/70	Immediate	BREAST-Q: 82/100 points in the questions about “overall satisfaction”, 81/100 points in the questions about “satisfaction with breasts”, the highest scores achieved in the questions about “satisfaction with the surgeon, medical staff and office staff”, lowest scores achieved in the questions about “sexual well-being”. Photos from 51/70 patients evaluated by three independent evaluators (surgeon, nurse, secretary): generally favorable outcome (mostly “good” or “very good”, agreement on breast projection, shape and volume, but disagreement in evaluation of breast symmetry and ptosis
2022, Francis et al. [22]	Nipple-sparing mastectomy (12/12)	1/12 patients in the control group and 5/12 patients in the intervention group with adjuvant chemotherapy or radiotherapy	Not reported	12/12 immediate reconstructions with monitoring skin paddle, 3/12 patients in the intervention group with delayed reconstruction	BREAST-Q: tend to be more satisfied in the intervention group (68/100 vs. 62/100 points in the questions about “satisfaction with breasts”) statistically significant difference only in the domain of “satisfaction with their surgeon” Manchester scar scale: Statistically significant difference in the domains “scar distortion”, “visual analogue scale” and “overall scar scale” favoring the intervention group
2023, Long et al. [23]	25/45 bilateral, 20/45 unilateral, 5/45 nipple-sparing, 40/45 type not reported	18/45 adjuvant	Not reported	Not reported	BREAST-Q: average score of 65/100 points in the domain “satisfaction with the breasts”, significantly higher score in patients with increased flap mass compared to the mastectomy mass (average difference in mass ~26.3%)
2023, Dung et al. [24]	Laterality not reported, radical modified mastectomy	Not reported	Not reported	Immediate	BREAST-Q: average score of 62/100 points in the questions about “satisfaction with the breasts”
